# Role and progression of bile acid metabolism in mediating Th17/Treg homeostasis in inflammatory bowel disease

**DOI:** 10.1016/j.isci.2026.114961

**Published:** 2026-02-09

**Authors:** Simei Yue, Yulin Tan, Lingjiao Gong, Fei Liao

**Affiliations:** 1 Department of Gastroenterology, Renmin Hospital of Wuhan University, Wuhan 430000, China; 2 Wuhan University Shenzhen Research Institute, Shenzhen, Guangdong 518000, China

**Keywords:** Health sciences, Medicine, Medical specialty, Proctology

## Abstract

Inflammatory bowel disease (IBD) is characterized by an aberrant immune response that results in intestinal inflammation and tissue damage, which manifest clinically as recurrent abdominal pain and diarrhea. As a chronic condition with limited curative options, the incidence of IBD has markedly increasing globally, imposing a considerable socioeconomic burden and leading to significant losses in workforce productivity. Recent research has indicated that the intestinal metabolite profile of patients with IBD is substantially altered compared to that of healthy individuals. Notably, a significant reduction was observed in the levels of microbiota-derived bile acid metabolites that are essential for gut immunity, indicating a potential association between bile acid metabolism and IBD progression. In this review, we aim to synthesize the role and recent research advancements regarding bile acid metabolites in mediating immune balance in IBD, thereby providing theoretical support for future investigations.

## Graphical abstract

**Figure:**
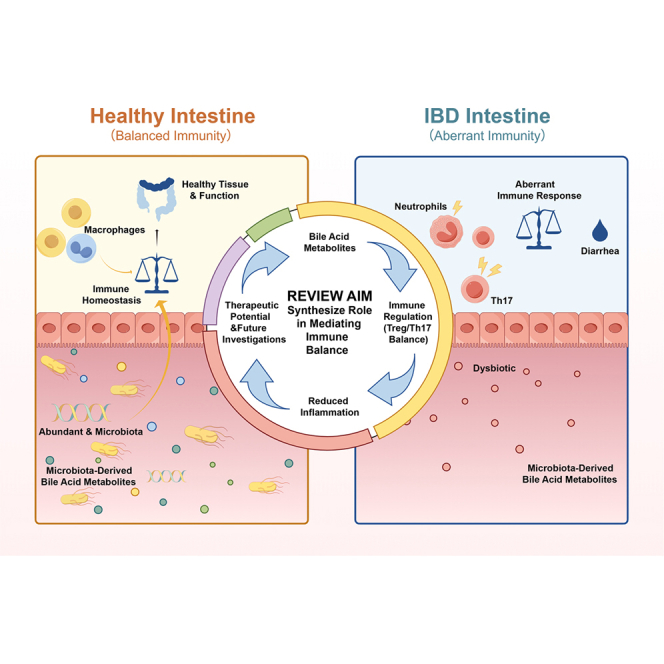

## Introduction

Inflammatory bowel disease (IBD) is a chronic immune-mediated disorder that predominantly affects the ileum, rectum, and colon, encompassing conditions such as ulcerative colitis (UC) and Crohn disease (CD). Recently, there has been a notable increase in the incidence and prevalence of IBD, which has garnered significant attention from the medical community.^1^ IBD is characterized by a challenging treatment course, high recurrence rate, and frequent association with severe complications, all of which substantially impair patients’ quality of life and impose considerable economic burdens on families and society. Due to its incurable nature, lifelong course, and potential for malignant transformation, IBD is often metaphorically referred to as a “green cancer.” However, the precise etiology and pathogenesis of IBD remain unclear. Increasing evidence from numerous studies suggests that dysbiosis of the gut microbiota and an imbalance in the intestinal immune system are critical factors influencing the progression of IBD.^2^ In individuals with IBD, the integrity of the intestinal barrier is compromised, facilitating the translocation of the intestinal flora into the intestinal wall. This translocation activates immune cells to produce cytokines, which further disrupt intestinal immune homeostasis. This disruption promotes the infiltration of additional immune cells into the intestinal wall and stimulates the body to mount an adaptive immune response. Activated immune cells can induce intestinal epithelial inflammation, compromise barrier function, and cause dysbiosis, ultimately resulting in chronic intestinal inflammation. Recent research has shown that metabolites derived from the intestinal microbiota, such as bile acids (BAs) and short-chain fatty acids (SCFAs), play regulatory roles in host intestinal immune function and metabolism.^3^ Furthermore, an increasing number of studies have demonstrated that the functional homeostasis of intestinal immune-associated helper T cells (Th17) and regulatory T cells (Tregs) is modulated by microbial metabolites that are crucial in the pathogenesis of IBD.^4^ This review summarizes the interaction between gut microbiota-derived BA metabolism and intestinal immune homeostasis in IBD. Our aim is to explore potential therapeutic approaches targeting the BA-gut microbiota axis for the treatment of IBD.

## Role of immune homeostasis in IBD

Immune homeostasis is a state in which the immune system maintains balance in the internal environment by regulating the inflammatory response in the body, protecting the host during pathogen attack and avoiding excessive damage to its tissues. Intestinal immune homeostasis is contingent upon the intricate interactions among immune cells, the intestinal microbiota, and intestinal epithelial cells. Disruption of this balance is critical for the pathogenesis of IBD.^5^

Immune cells secrete cytokines to regulate the inflammatory response, but they may mistakenly attack their own tissues when targeting harmful substances, such as pathogens, thereby exacerbating chronic inflammation. This dysregulated immune response induces a systemic inflammatory reaction in the intestinal tract, manifesting as abdominal pain and diarrhea, which significantly diminishes a patient’s quality of life.^6^ Th17 cells are a subpopulation of T cells that can be differentiated from naive CD4^+^ T cells. Their primary characteristic is the secretion of interleukin (IL)-17, although they also produce other pro-inflammatory cytokines, such as IL-21, IL-22, and IL-23, which collectively contribute to the exacerbation of intestinal inflammation.^7^ Ohara et al. reported significantly elevated levels of IL-17 specifically secreted by Th17 cells in the intestinal mucosa and serum of patients with active IBD, suggesting that Th17 cells play a crucial role in IBD pathogenesis.^8^ In contrast to Th17 cells, Tregs represent a subpopulation of CD4^+^ T cells with immunomodulatory functions that are capable of controlling the progression of intestinal inflammation by exerting negative immunomodulatory effects, suppressing immune responses, and promoting immune tolerance.^9^ Mowat et al. demonstrated that intestinal macrophages produce IL-10, which mitigates excessive inflammatory responses and enhances immune tolerance in the gut by upregulating CD4^+^CD25^+^ Tregs.^10^ However, Acharya et al. observed a significant reduction in the number of Tregs in the peripheral blood of mice with experimental colitis.^11^ Enhancing the secretion of Treg-associated cytokines, such as IL-10 and transforming growth factor β (TGF-β), along with other anti-inflammatory factors, has been shown to ameliorate diarrheal symptoms in mice with IBD.^12^

Thus, microbiota metabolism-mediated abnormalities in the intestinal immune system caused by an imbalance in Th17/Treg cells play a key role in the onset, development, and prognosis of IBD.

## ba metabolic pathways and organismal immune homeostasis

**Figure 1:**
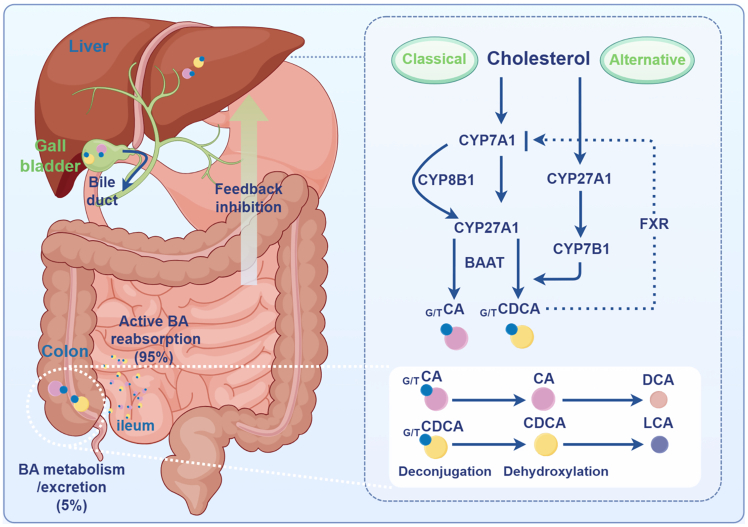
Synthesis of bile acids in the body Cholesterol is converted to primary bile acids (CA and CDCA) via classical (CYP7A1-initiated) or alternative (CYP27A1-initiated) pathways. After conjugation with glycine or taurine (G/T), bile acids are secreted into the intestine. Approximately 95% are actively reabsorbed in the ileum and return to the liver, completing the cycle; the remaining 5% undergo gut bacterial deconjugation and dehydroxylation to form secondary bile acids (DCA and LCA). The nuclear receptor FXR provides negative feedback by inhibiting CYP7A1, regulating bile acid homeostasis. CA, cholic acid; CDCA, chenodeoxycholic acid; DCA, deoxycholic acid; LCA, lithocholic acid; CYP7A1, cholesterol 7α-hydroxylase; CYP27A1, sterol-27-hydroxylase.

BAs are synthesized from cholesterol in the liver and released into the duodenum after the ingestion of food, which, in turn, facilitates the digestion and absorption of lipids in the body (Figure 1). BAs can be divided into primary bile acids (PBAs) and secondary bile acids (SBAs). PBAs are primarily produced via the classical pathway, which is mediated by cholesterol 7α-hydroxylase (CYP7A1), and the alternative pathway, mediated by sterol-27-hydroxylase (CYP27A1). The main PBAs are cholic acid (CA) and chenodeoxycholic acid (CDCA).

Following their synthesis, PBAs are conjugated to glycine or taurine and excreted into the intestine. At the terminal ileum, approximately 95% of BAs are reabsorbed into enterocytes via the apical sodium-dependent BA transporter (ASBT) and enter the enterohepatic circulation. The remaining PBAs are further metabolized by the intestinal flora. Conjugated BAs are deconjugated and converted by intestinal flora to SBAs such as deoxycholic acid (DCA), lithocholic acid (LCA), and ursodeoxycholic acid (UDCA) by 7α-dehydroxylation and epimerization. Most BAs in the aforementioned process are reabsorbed by the body in the distal small intestine, with the residual BAs entering the colon at concentrations ranging from approximately 200 to 1000 μM. A portion of these BAs is subsequently excreted in feces.^13^

**Figure 2:**
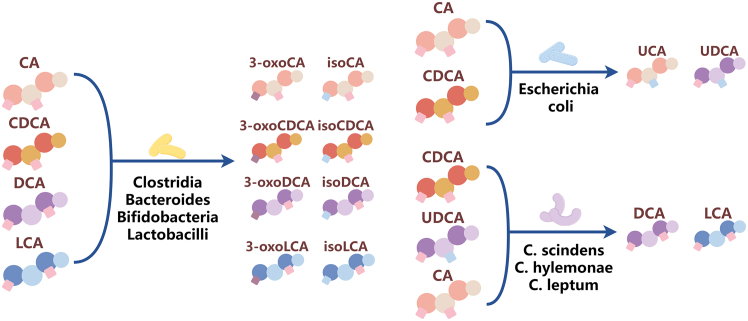
Bacterial taxa contributing to bile acid metabolism Key bacterial taxa sequentially modify primary bile acids (CA and CDCA) through deconjugation, dehydrogenation, and dehydroxylation reactions, producing immunomodulatory secondary bile acids (e.g., DCA, LCA, and iso-LCA) that crucially influence host immune homeostasis in IBD. CA, cholic acid; CDCA, chenodeoxycholic acid; DCA, deoxycholic acid; LCA, lithocholic acid.

BAs possess stable structures characterized by four rings and a hydroxyl group at the C-3 position. The predominant BAs found in the human gallbladder exhibit hydroxylation on the rings at specific positions, with stereochemistry at 3α, 7α, and 12α, and are conjugated with the amino acids taurine or glycine at the side chain carboxylic acid. Subsequently, the microbiome modifies these BAs through various biochemical processes (Figure 2). Furthermore, when BAs are released into the environment, they are biotransformed by microorganisms, leading to the formation of distinct metabolites that retain only the C and D rings of the original BA structure (Table 1).^14^

**Table 1:** Physiological and pathological effects of various bile acid metabolites

Abbreviation	Bile acid name	Pathophysiologic functions
CA	cholic acid	facilitates fat absorption and cholesterol excretion^110^
CDCA	chenodeoxycholic acid	reduces cholesterol saturation in bile and dissolves gallstones^111^
GCA	glycocholic acid	inhibits LPS-induced macrophage recruitment and pro-inflammatory factor secretion^112^
GCDCA	glycochenodeoxycholic acid	1. inhibits autophagosome formation and impairs lysosomal function by inhibiting lysosomal protein hydrolysis and increasing lysosomal pH in normal human hepatocytes, leading to apoptosis of human hepatocytes 2. induces stem cell properties and chemoresistance in hepatocellular carcinoma cells through activation of the STAT3 signaling pathway^113^^,^^114^
TCA	taurocholic acid	inhibits LPS-induced macrophage recruitment and pro-inflammatory factor secretion^112^
DCA	deoxycholic acid	promotes fat absorption and cholesterol excretion^115^
UDCA	ursodeoxycholic acid	exerts protective effect against neurodegeneration in an animal model^116^
HDCA	hyodeoxycholic acid	significantly increases abundances of probiotic species such as *Parabacteroides distasonis*, which enhance lipid catabolism through fatty acid-hepatic peroxisome proliferator-activated receptor alpha (PPARα) signaling^117^
GDCA	glycodeoxycholic acid	induces hepatocyte necrosis and autophagy in patients with obstructive cholestasis^13^
GUDCA	glycoursodeoxycholic acid	modulates bile acid levels and alters gut microbiota and glucolipid metabolism, thereby easing the progression of diabetes mellitus^118^
LCA	lithocholic acid	at high concentrations, induces oxidative stress and DNA damage and promotes tumor development by inhibiting DNA repair enzymes and promoting cell proliferation^119^
GLCA	glycolithocholic acid	alters intestinal flora composition and modulates the intestinal inflammatory response in ulcerative colitis^120^

BAs originate from cholesterol oxidation and are conjugated. BAs undergo modifications within the digestive tract, resulting in a diverse array of compounds. These BAs, which originate from cholesterol synthesis and bacterial metabolism, collectively form a comprehensive BA network. Recent advancements of analytical techniques such as high-performance liquid chromatography-mass spectrometry have elucidated the structures of tens of thousands of distinct BAs derived from the intestinal microbiota. DCA was the first hydrophobic BA product identified in the intestinal flora, and its distinct hydrophobic properties have garnered significant attention because of its potential toxicity to bacteria and enterocytes.^15^

BAs are intricately linked to various biological functions and have been demonstrated to influence the progression of inflammatory diseases including IBD and asthma. Within the immune system, BAs inhibit the production of inflammatory factors such as IL-1β and IL-6 by immune cells and attenuate the body’s inflammatory response.^16^ In addition, BAs are essential for regulating metabolic homeostasis by activating thyroid hormones, which, in turn, promote energy expenditure in brown adipose tissues.^17^ Notably, the concentration of SBAs in feces was lower in patients with IBD than in healthy individuals, indicating a possible impairment in the dissociation and transformation of BAs in the gut.^18^ Sinha et al. conducted BA profiling to examine the percentage of BAs in patients with UC and familial adenomatous polyposis. Their findings revealed a significant reduction in SBAs, such as LCA and DCA, in the serum of patients with UC, whereas the levels of CDCA were found to be increased. They also found that SBAs attenuated the progression of inflammation in a mouse model of colitis while downregulating the expression of key pro-inflammatory factors in intestinal inflammation. Meanwhile, research in a mouse model of colitis has demonstrated that LCA and DCA can mitigate inflammation. This is evidenced by reduced weight loss, a decreased disease activity index, and diminished structural damage to the colon.^19^ Additionally, the introduction of the intestinal flora from patients with IBD-induced intestinal immune disorders into germ-free mice has been reported. This induction was characterized by an increase in pro-inflammatory factors, including IL-17, IL-22, and Th17 cells, and a decrease in anti-inflammatory factors such as IL-10 and TGF-β.^20^ Furthermore, the BA derivative isoalloLCA inhibited Th17 expression, while promoting the differentiation of naive CD4^+^ T cells into Tregs. This modulation of the Th17/Treg cell balance can attenuate the intestinal inflammatory cascade, thereby alleviating the clinical symptoms of IBD.^21^^,^^22^ These findings suggest that various factors, including the intestinal microbiota and its metabolites, collectively influence Th17/Treg cellular equilibrium.

In a previous study, BAs exerted anti-inflammatory effects by modulating the intestinal Treg/Th17 cell balance, thereby decelerating the progression of IBD.^16^ It is evident that BA metabolism plays an important role in intestinal intrinsic immunity, and SBAs have received more attention in IBD. An increasing number of studies have confirmed that SBAs delay IBD progression by inhibiting inflammatory responses.

## Pathways of BAs in IBD

BA-activated receptors are a class of receptors that can be specifically stimulated by BAs because of their physiological activities. These receptors include nuclear receptors (NRs) and G-protein-coupled receptors (GPCRs), which play important roles in BA synthesis and function. Among them, the farnesoid X receptor (FXR), pregnane X receptor (PXR), and vitamin D receptor (VDR) are NRs, and G protein-coupled BA receptor 1 (GPBAR1) is a GPCR. BA-activated receptors regulate embryogenesis, organismal development, and immune regulation and play an important role in the progression of IBD.

### FXR

**Figure 3:**
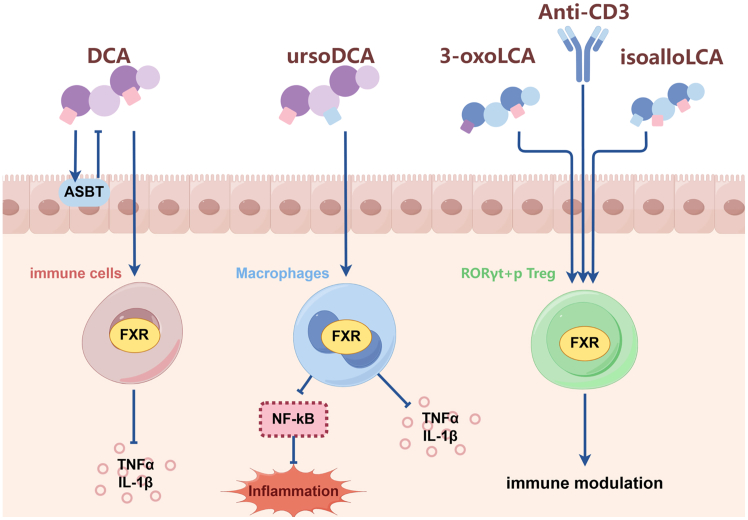
Bile acids interact with FXR to regulate IBD progression ursoDCA activates FXR in macrophages, inhibiting NF-κB and thereby suppressing inflammatory cytokines (e.g., TNF-α and IL-1β). Conversely, DCA enters immune cells via the ASBT to activate FXR, potentially exacerbating inflammation. Secondary bile acids 3-oxoLCA and isoalloLCA activate FXR in RORγt^+^ pTreg cells, promoting a tolerogenic response. ursoDCA, ursodeoxycholic acid; DCA, deoxycholic acid; ASBT, apical sodium-dependent bile acid transporter; 3-oxoLCA, 3-oxo-lithocholic acid; RORγt^+^, retinoic acid-related orphan receptor gamma t.

FXR serves as the central regulatory node for systemic BA homeostasis. It is predominantly expressed in the liver and intestine and exhibits a hierarchy of ligand specificity, with CDCA as its most potent endogenous agonist, followed by DCA, LCA, and CA (Figure 3). Upon ligand binding, FXR initiates a sophisticated transcriptional cascade. A cornerstone of this response is the transactivation of the small heterodimer partner (SHP), which, in turn, competes with and displaces HNF4α from the promoter of the rate-limiting enzyme CYP7A1, establishing a critical autoregulatory loop for BA synthesis.^23^ This NR also orchestrates BA transport, enhancing biliary excretion via the bile salt export pump (BSEP) while inhibiting basolateral uptake through the sodium taurocholate co-transporting polypeptide, thereby preventing cytotoxic BA accumulation.^24^ The physiological relevance of this intricate regulation is underscored by studies showing that *FXR* knockout in mice leads to profoundly disrupted BA homeostasis and systemic metabolic dysregulation.^25^

A defining feature of FXR biology is its role in mediating enterohepatic crosstalk. Intestinal FXR activation stimulates the production of fibroblast growth factor 19 (FGF19 in mice), which, upon release into the portal circulation, activates hepatic FGFR4/β-Klotho receptor complexes. This endocrinological axis suppresses CYP7A1 expression via an ERK1/2 phosphorylation cascade, providing a refined remote control over hepatic BA production.^26^ Within the intestinal compartment itself, FXR activation inhibits ASBT, thereby modulating BA enterohepatic circulation and protecting enterocytes from potential BA overload.^27^

Beyond its metabolic functions, FXR exerts potent anti-inflammatory and barrier-protective effects highly relevant to IBD pathophysiology. Its activation suppresses pro-inflammatory signaling through multilayered mechanisms, including the direct sequestration and promotion of proteasomal degradation of the NF-κB p65 subunit, and the transcriptional repression of cytokines like TNF-α, IL-6, and IL-1β via chromatin-bound SHP complexes.^28^ Consequently, FXR activation has been demonstrated to ameliorate intestinal inflammation in murine colitis models. For instance, UDCA was shown to interact with macrophage FXR to inhibit NF-κB activation and cytokine production,^29^ while other studies confirm that FXR activation downregulates mucosal pro-inflammatory cytokines, reduces epithelial permeability, and mitigates colitis symptoms.^25^

Furthermore, FXR plays an integral role in maintaining intestinal barrier integrity by stabilizing tight junction proteins (e.g., claudin-1 and occludin) and stimulating the secretion of antimicrobial peptides. Its influence also extends to immune cell differentiation, where it helps maintain the balance between Th17 and Tregs cells. This is exemplified by the finding that specific BAs like 3-oxo-lithocholic acid (3-oxoLCA) and isoalloLCA can modulate the differentiation of Th17 and peripherally derived Treg (pTreg) cells in an FXR- and VDR-dependent manner, thereby exerting a protective effect in colitis models.^21^

The therapeutic potential of targeting this receptor is underscored by the discovery of novel regulatory components, such as the liver-specific enhancer RNA Fincor, which is essential for the resolution of steatohepatitis by the FXR agonist tofexiflex. Given its central role in coordinating BA metabolism, inflammation, and barrier function, FXR emerges as a promising and multifaceted therapeutic target for intervening in the progression of IBD.^30^

### GPBAR1

**Figure 4:**
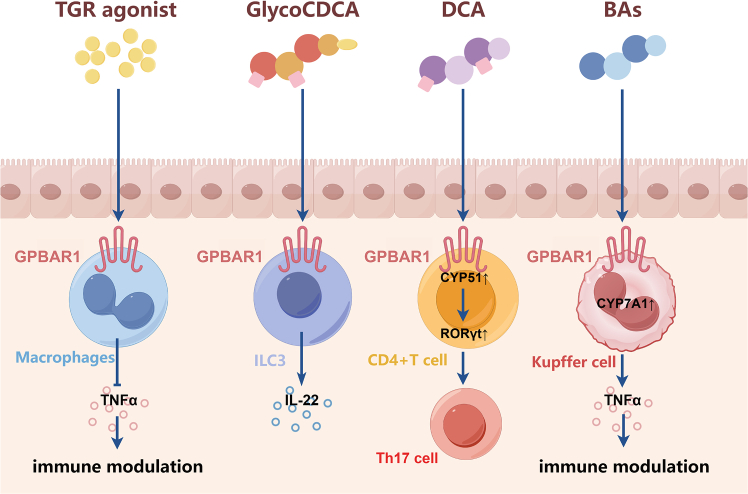
Bile acid binding to GPBAR1 regulates the immune and inflammatory responses through different mechanisms TGR5 agonists suppress TNF-α in macrophages, DCA promotes IL-22 in Group 3 Innate Lymphoid Cells (ILC3s), and BA binding upregulates CYP51/RORγt to promote Th17 differentiation in CD4^+^ T cells.

GPBAR1, commonly known as TGR5, represents a pivotal GPCR that functions as a metabolic and immunological sensor for BAs (Figure 4). While initially characterized for its role in energy expenditure and glucose metabolism, its widespread expression in the intestinal compartment, such as L-cells, enteric neurons, myofibroblasts, and diverse immune subsets, position it as a central regulator of the gut-liver axis. GPBAR1 is activated primarily by LCA and DCA, serving as a molecular bridge that translates microbial metabolic output into host physiological responses.^31^

The most extensively documented role of GPBAR1 in IBD pathology lies in its potent immunomodulatory capacity within the innate immune system, particularly in monocytes and macrophages. Upon ligand binding, GPBAR1 couples with the Gαs protein, triggering the activation of adenylyl cyclase and a subsequent surge in intracellular cAMP.^32^ This second messenger is the linchpin of GPBAR1-mediated immunosuppression. Mechanistically, elevated cAMP activates protein kinase A, which phosphorylates the cAMP-response element-binding protein. More critically, protein kinase A activation interferes with the NF-κB signaling pathway.^33^ In the absence of GPBAR1 activation, inflammatory stimuli induce the phosphorylation and degradation of IκBα, allowing the p65/p50 NF-κB complex to translocate to the nucleus and drive pro-inflammatory gene expression. GPBAR1 activation inhibits this translocation. Pols et al. and Perino et al. have elucidated that this inhibition effectively blocks the transcription of key cytokines, including TNF-α, IL-1β, IL-6, and MCP-1.^34^ Thus, *GPBAR1*-knockout mice exhibit increased susceptibility to colitis, characterized by abnormal mucosal morphology and elevated inflammatory mediators.^35^^,^^36^

Beyond mere cytokine suppression, GPBAR1 acts as a molecular switch regulating macrophage plasticity. In the context of colitis, intestinal macrophages often adopt a pro-inflammatory M1 phenotype, contributing to tissue destruction. Activation of GPBAR1 has been shown to skew this differentiation toward an anti-inflammatory M2 phenotype, characterized by the expression of arginase-1 and CD206 and the secretion of the resolution-phase cytokine IL-10.^37^ This M1-to-M2 shift is essential for the resolution of inflammation and mucosal healing, as demonstrated in GPBAR1-deficient mice, which exhibited exacerbated colitis severity, delayed recovery, and persistent M1 macrophage infiltration compared to wild-type littermates.^38^

Although NF-κB suppression is important, investigations over the last decade have emphasized the role of NLRP3 inflammasome regulation in GPBAR1 function. The NLRP3 inflammasome is a multiprotein complex that processes pro-IL-1β and pro-IL-18 into their active forms. Dysregulated NLRP3 activity is a hallmark of severe IBD. Fiorucci et al. and Guo et al. have established GPBAR1 as a negative regulator of this complex.^38^^,^^39^ Specifically, the cAMP-protein kinase A signaling cascade initiated by GPBAR1 leads to the phosphorylation of NLRP3 at specific serine residues, which ubiquitylates NLRP3 and targets it for proteasomal degradation or prevents its assembly. Studies in models of sterile inflammation and chemically induced colitis have indicated that the absence of GPBAR1 removes this negative regulation resulting in uncontrolled inflammasome activation and pyroptosis, thereby amplifying the inflammatory cascade.^39^ In models of liver injury, GPBAR1 deficiency exacerbates inflammation by promoting NLRP3 activation, caspase-1 cleavage, and subsequent IL-1β and IL-18 production, thereby driving pro-inflammatory M1 macrophage polarization.^40^^,^^41^^,^^42^ These findings underscore that GPBAR1 operates on transcriptional and post-translational mechanisms to maintain immune tolerance.

The integrity of the intestinal epithelial barrier is paramount in preventing the translocation of luminal pathogens. While earlier research has focused heavily on immune cells, accumulating evidence points to a direct protective role of GPBAR1 in epithelial cells. A critical factor in epithelial dysfunction in IBD is endoplasmic reticulum (ER) stress (ERS). Unresolved ERS triggers the unfolded protein response (UPR), leading to epithelial cell apoptosis and barrier leakage. Recent studies suggest that BAs, via GPBAR1, can modulate this stress response. Activation of GPBAR1 has been linked to the attenuation of the IRE1α-JNK signaling arm of the UPR, a pathway known to drive apoptosis and inflammation.^43^ By reducing ERS, GPBAR1 helps preserve the function of goblet cells and Paneth cells, ensuring adequate mucus production and antimicrobial peptide secretion.^44^ Furthermore, GPBAR1 signaling reinforces the physical barrier. It promotes the expression and proper localization of tight junction proteins.^44^

Despite the overwhelming evidence supporting an anti-inflammatory role, the receptor’s function is highly dependent on the local concentration of BAs and the specific cell type involved. For instance, while GPBAR1 activation on macrophages is protective, its activation on inhibitory motor neurons in the colon can promote motility, which, in dysbiotic conditions, might contribute to diarrhea, a common symptom of IBD.^45^ Furthermore, systemic activation of GPBAR1 is known to cause side effects such as gallbladder filling and severe pruritus, mediated by GPBAR1 expression on sensory neurons.^46^ This has historically hampered the clinical development of systemic GPBAR1 agonists. Moreover, the interaction between GPBAR1 and adaptive immunity is complex. While some studies suggest that GPBAR1 promotes the secretion of protective IL-22 via lymphoid cells,^47^ others indicate that under high-fat diet conditions, elevated DCA levels can stimulate pathways that indirectly support Th17 differentiation, potentially exacerbating autoimmunity in specific genetic backgrounds.^21^ Current therapeutic strategies are, thus, shifting toward intestine-restricted GPBAR1 agonists that harness the anti-inflammatory and barrier-protective benefits while minimizing systemic adverse effects.

This collective evidence positions the intestinal microbiota-BA-GPBAR1 axis as a critical regulator of intestinal barrier function and inflammation, making GPBAR1 a compelling therapeutic target for modulating immune homeostasis in IBD.

### PXR

PXR is a member of the orphan NR abundantly expressed in the liver and intestine.^48^ Initially identified as the key regulator of cytochrome P450 3A4 (CYP3A4), PXR forms a heterodimer with the retinoid X receptor to control a network of genes involved in drug and endobiotic metabolism.^49^ Its role, however, extends far beyond detoxification, encompassing critical functions in intestinal inflammation, barrier integrity, and cancer.^50^

A pivotal aspect of PXR’s function is its interaction with gut microbiome-derived metabolites. SBAs, particularly LCA and DCA, serve as endogenous PXR ligands. This activation induces hydroxylating enzymes like Cyp3a11, providing a protective mechanism against BA-induced hepatotoxicity.^51^ Furthermore, the microbial tryptophan metabolite indole-3-propionic acid (IPA) is a potent intestine-specific PXR ligand. IPA activation strengthens the gut barrier by upregulating tight junction proteins and suppressing pro-inflammatory cytokines like TNF-α, thereby mitigating intestinal inflammation. The physiological importance of this pathway was highlighted in PXR-deficient mice, which exhibited a “leaky” gut phenotype and heightened sensitivity to inflammatory insults.^52^

Novel research reveals a metabolic role for intestinal PXR activation. The intestinal-selective agonist tributyl citrate was shown to counteract high-fat diet-induced obesity in mice. This beneficial effect is attributed to the upregulation of β-1,3-galactosyltransferase 5, a key mediator through which PXR activation strengthens the intestinal barrier and improves metabolic homeostasis.^53^

PXR is expressed throughout the intestinal tract and plays a protective role against IBD through a key mechanism of mutual antagonism with the NF-κB signaling pathway. On the one hand, pharmacological activation of PXR suppresses the production of inflammatory mediators from intestinal epithelial cells.^54^ On the other hand, the p65 subunit of NF-κB can bind to RXRα, the heterodimeric partner of PXR, thereby inhibiting PXR’s transcriptional activity.^55^ This bidirectional inhibitory mechanism forms a critical inflammatory regulatory hub, and its disruption exacerbates intestinal inflammation. In addition to this crosstalk, PXR activation in macrophages has been shown to promote caspase-1-dependent IL-1β release via an NLRP3 inflammasome-dependent pathway.^56^

Targeting PXR therapeutically is an emerging frontier. The development of FKK6, a direct PXR-binding ligand designed to mimic the microbial metabolite IPA, demonstrates this potential. FKK6 exhibits anti-inflammatory effects in models of humanized PXR expression and shows promise in suppressing colitis-associated colon cancer.^57^

PXR serves as a master regulator of intestinal homeostasis, forming a critical functional bridge between xenobiotic metabolism, immune responses, and the gut microbiome. Furthermore, microbiota-derived metabolites like IPA are central to PXR’s function, opening new paths for treating inflammatory and metabolic diseases. Moving forward, research must prioritize clarifying the molecular details of these interactions and harnessing the translational potential of PXR-targeted therapies. A deeper understanding of these networks is essential for pioneering personalized treatment modalities in gastroenterology.

### VDR

The VDR functions as a nuclear transcription factor with extensive physiological implications and is ubiquitously expressed in cells across various tissues, including the human intestine, liver, and pancreas. It primarily plays a role in the regulation of diseases affecting the intestine, liver, skin, and other organs. Vitamin D derivatives, SBAs, and bile salts serve as ligands that activate the VDR.^58^ The pathogenesis of IBD is associated with diminished intestinal microbiota, aberrant inflammatory responses, and deficiencies in micronutrients such as vitamin D. Notably, severe vitamin D deficiency significantly increases the risk of developing IBD.^59^ In a randomized, double-blind, placebo-controlled trial, daily oral supplementation with 1,200 IU of vitamin D3 significantly increased serum vitamin D levels and reduced the risk of recurrence from 29% to 13% in patients with CD.^60^

Additionally, VDR expression in the colon of patients with IBD has been reported to be inversely correlated with the histological scores of IBD, suggesting a protective role for VDR against intestinal inflammation.^61^ Zhu et al. showed that VDR inhibits apoptosis in intestinal epithelial cells by downregulating p53 expression.^62^ Thus, VDR overexpression in mice attenuates colitis while maintaining the intestinal epithelial barrier.^63^ Recent studies have demonstrated that the absence of VDR *in vivo* leads to a decreased abundance of colonic Tregs, indicating a crucial role for VDR in maintaining intestinal Treg cell homeostasis.^64^ These findings highlight the beneficial role of the VDR in modulating intestinal inflammation.

Furthermore, the regulation of retinoic acid-related orphan receptor gamma t (RORγt^+^) Tregs by VDR is intimately linked to the pathogenesis of IBD. Mutations in VDR, which are associated with IBD, can modulate disease susceptibility by affecting the regulation of intestinal Tregs. Particularly, the intestinal Treg transcription factor, VDR, may regulate the balance of colonic Tregs by coordinating BA signaling with transcription factor activity. Therefore, further understanding of the molecular mechanisms by which the BA signaling network between the host and its associated microbes regulates intestinal Tregs would be valuable for improving the treatment of inflammatory diseases of the human gastrointestinal tract.

### RORγt

RORγt acts as the master transcriptional regulator for Th17 cells. Historically, RORγt was viewed primarily as a sensor of sterol intermediates in the cholesterol biosynthesis pathway.^65^ However, it has been reported, in recent years, that RORγt serves as a direct NR for specific microbial-derived SBAs, indicating that the metabolic output of the microbiome directly interfaces with the host’s adaptive immune system to dictate the balance between inflammation and tolerance.^21^

The molecular basis for this interaction lies in the structural architecture of the RORγt ligand-binding domain (LBD). Structural biology studies utilizing X-ray crystallography have revealed that the LBD contains a hydrophobic pocket that is canonically occupied by endogenous oxysterols, which stabilize the receptor in an active conformation to drive pro-inflammatory gene transcription.^65^ Crucially, specific BA metabolites, including 3-oxoLCA, possess a steroid scaffold that allows them to competitively bind to this pocket.^21^ Unlike endogenous agonists, 3-oxoLCA functions as a specific inverse agonist. Upon binding, it induces a conformational change in helix 12 of the LBD.^66^ This structural shift displaces transcriptional co-activators and promotes the recruitment of co-repressors like Nuclear receptor corepressor (NCOR).^67^ This transcriptional repression directly silences the expression of the hallmark cytokines IL-17a, thereby inhibiting the potential of Th17 cells to cause inflammation. Furthermore, RORγt expression is not exclusive to Th17 cells; it also defines a specialized subset of colonic Treg cells known as RORγt^+^ Tregs. This subpopulation is uniquely adapted to the intestinal microenvironment and exhibits superior anti-inflammatory properties compared with conventional Tregs. Song et al. demonstrated that the maintenance of this RORγt^+^ Treg pool is strictly dependent on BA signaling. In the absence of specific BA ligands, these cells lose their suppressive capacity and can transdifferentiate into pro-inflammatory Th17-like cells, exacerbating colitis.^68^

The clinical relevance of the RORγt-BA axis in IBD is profound. Metagenomic and metabolomic analyses have consistently shown that patients with active IBD exhibit a depletion of specific bacterial species capable of converting PBAs into these immunomodulatory metabolites.^69^ Consequently, the intestinal tissue of IBD patients is deficient in these natural RORγt inverse agonists, leading to unchecked RORγt activity and chronic Th17-driven inflammation.^70^ Therefore, the therapeutic approach targeting the RORγt-BA interface offers a precision medicine solution, dampening the autoimmune components of IBD while preserving protective immunity.

## BA metabolism and ERS

The ER is a key cellular organelle responsible for protein folding and processing. Disruption of ER homeostasis triggers ERS, leading to activation of the UPR to restore cellular function.^71^ Emerging evidence indicates a close bidirectional regulatory relationship between BA metabolism and ERS, a mechanism particularly relevant to the pathogenesis of IBD.

At the molecular level, hydrophobic BAs such as DCA, at pathological concentrations, can induce ERS and mitochondrial dysfunction in both hepatocytes and intestinal epithelial cells, thereby promoting inflammatory responses. In contrast, hydrophilic BAs like UDCA exhibit protective effects and can ameliorate chemically induced ERS.^72^ Studies have confirmed that ERS upregulates immunoglobulin-binding protein and the transcription factor CHOP, which, in turn, suppresses the expression of key BA synthesis enzyme CYP7A1 and impairs the function of the BA transporter BSEP, ultimately altering BA composition.^73^ Notably, gut-specific ERS can remotely regulate hepatic BA synthesis via the gut-liver axis; for example, mice lacking XBP1 are more susceptible to BA dysregulation and liver injury.^74^

In the context of IBD, sustained ERS activation in intestinal epithelial cells leads to impaired antimicrobial peptide secretion, epithelial barrier dysfunction, and apoptosis, collectively exacerbating intestinal inflammation.^75^ Clinical data show elevated expression of ERS markers such as GRP78 in the intestinal epithelium of IBD patients.^76^ ERS also influences BA signaling by modulating the expression of FXR and PXR. Experimental studies have demonstrated that the inhibition of ERS improves BA metabolic disturbances and alleviates intestinal inflammation in IBD models.^77^

Current research suggests that gut microbiota-derived BA metabolites may serve as important regulators of ERS. For instance, *Odoribacter* species have been shown to ameliorate colitis by suppressing the NF-κB pathway via ERS modulation.^78^ However, the precise mechanisms by which specific BA species regulate distinct UPR signaling branches such as the IRE1α-XBP1 and PERK-ATF4 pathways, and how this regulation dynamically changes during different stages of IBD remain to be fully elucidated. Clarifying these mechanisms may provide new insights into therapeutic strategies that simultaneously target BA metabolism and ERS in IBD.

## Intestinal flora modulates BA conversion in IBD

**Figure 5:**
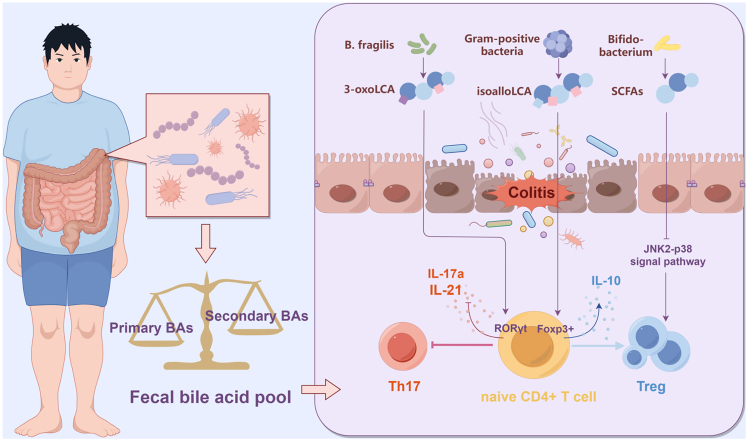
Intestinal flora-derived bile acids modulate immune homeostasis in IBD The intestinal microbiota (e.g., *B. fragilis*, Gram-positive bacteria, and *Bifidobacterium*) biotransform primary bile acids and other precursors into immunomodulatory metabolites, including secondary bile acids (3-oxoLCA and isoalloLCA) and short-chain fatty acids (SCFAs). 3-oxoLCA acts as an RORγt inverse agonist to potently inhibit Th17 differentiation and the production of IL-17a/IL-21; isoalloLCA and SCFAs promote the differentiation of anti-inflammatory Treg cells expressing Foxp3 and secreting IL-10. 3-oxo-lithocholic acid; RORγt^+^, retinoic acid-related orphan receptor gamma t.

Approximately 5%–10% of BAs are secreted into the colon, where they undergo biotransformation by the intestinal microbiota or are excreted in feces (Figure 5). The intestinal microbiota influences the expression of *CYP7A1*, *CYP7B1*, and *CYP27A1* in mice, thereby regulating BA metabolism. Specific bacterial species, including *Gordonibacter pamelaeae*, *Eggerthella lenta*, *Ruminococcus gnavus*, and *Clostridium scindens,* have been demonstrated to metabolize PBAs into SBAs through the production of the BA 7α-dehydroxylases.^15^ Duboc et al. observed that dysbiosis, resulting from compromised enzymatic activity in gut microorganisms, may further modulate the composition of the BA pool in the gut lumen.^70^ In addition, BAs affect the composition of microorganisms. On the one hand, high concentrations of BAs induce DNA and oxidative damage in bacteria,^79^ and on the other hand, BAs indirectly influence bacterial growth by binding to receptors such as FXR and VDR.^80^ Patients with IBD exhibit a reduction in the overall abundance of gut microbiota, including a notable decline in beneficial microbial populations, such as those belonging to the phylum Firmicutes. Concurrently, an increase in the number of pathogenic microorganisms is observed, including members of the genus *Aspergillus* and *Escherichia coli*.^81^ In patients with UC, a significant reduction in SCFA-producing bacteria, specifically *Bacillus pumilus*, is observed.^82^ Recent research has demonstrated that the oral administration of BAs can modify the composition of BAs and the gut microbiota *in vivo*. Xu et al. demonstrated that the oral administration of DCA to mice resulted in a significant reduction in the abundance of intestinal microbiota, accompanied by an increase in the prevalence of specific strains, such as *Clostridium difficile* and *Ehrlichia*, ultimately culminating in intestinal inflammation.^83^ Thus, BAs and other metabolites produced by gut microbiota concurrently influence the microbiota. This observation implies that therapeutic strategies involving microbiota transplantation should be integrated with protocols aimed at rebalancing metabolites of the intestinal flora to prevent dysregulation of the intrinsic metabolic environment of the transplanted microbiota.

## Mutual regulation of intestinal flora and BA metabolism co-regulate immune homeostasis in IBD

**Figure 6:**
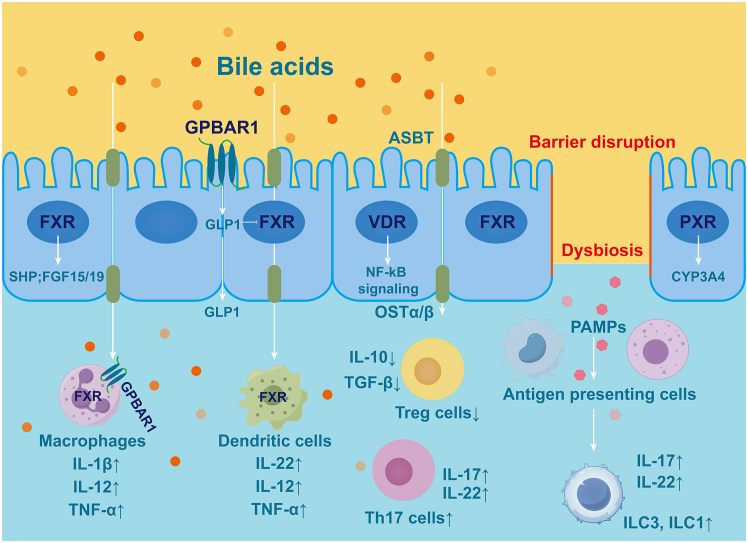
Schematic of receptor-mediated gut microbiota-bile acid axis dysregulation in intestinal inflammation In the intestinal barrier, disrupted epithelial cells show impaired signaling through receptors (FXR, PXR, VDR, and GPBAR1), leading to barrier dysfunction. In the lamina propria, this facilitates PAMP-driven activation of antigen-presenting cells (e.g., macrophages and dendritic cells), skewing the immune response toward a pro-inflammatory state with elevated Th17/ILC3-derived cytokines (IL-17 and IL-22) and reduced Treg-associated mediators (IL-10 and TGF-β).

The imbalance between Th17 and Treg cells is a critical factor in the pathogenesis and progression of IBD (Figure 6). Therefore, modulating the balance between Th17 and Treg cells is a central concept in managing the progression of IBD. Previous studies have demonstrated that patients with IBD exhibit an increased presence of Th17 cells, which secrete pro-inflammatory cytokines such as IL-17 and IL-22, alongside a reduction in Treg cells, which produce anti-inflammatory cytokines like IL-10 and TGF-β, within the gut. This imbalance contributes to an inflammatory milieu and influences the composition of the gut microbiome.^4^

Hang et al. found that 3-oxoLCA, derived from *Bacteroides fragilis*, inhibits the differentiation of Th17 cells through interaction with RORγt.^84^ Additionally, Foxp3-expressing Tregs are crucial for mitigating intestinal inflammatory responses.^85^ Metabolites produced by the gut flora, including BAs, have been shown to induce peripheral Treg production. SCFAs derived from *Bifidobacterium* inhibit JNK1 and p38 signaling pathways, thereby promoting the proliferation of intestinal Tregs.^86^ Song et al. reported that the downregulation of the BA metabolic pathway significantly decreased the number of Treg cells in the intestine.^87^ In contrast, a notable increase in Treg cell numbers was observed following restoration of BA metabolism.^64^ The binding of *Faecalibacterium*-derived butyrate to the GPR109A receptor in the colon enhances anti-inflammatory properties of colonic macrophages and dendritic cells and induces Treg differentiation.^88^ A recent study indicated that isoalloLCA, derived from certain gram-positive bacteria in the intestine, facilitates the differentiation of anti-inflammatory Tregs by promoting the formation of chromatin structures in the Foxp3 promoter region. Furthermore, the concentrations of isoalloLCA and the prevalence of isoalloLCA-producing bacteria were significantly lower in patients with IBD than in healthy controls. Moreover, oral administration of isoalloLCA to IBD-afflicted mice enhanced Treg activity in the intestinal lamina propria, thereby mitigating disease progression.^89^

These studies highlight the potential of therapeutic strategies aimed at modulating Treg cell function by using BA metabolites. Such approaches may sensitize Treg cells to precisely regulate intestinal immune homeostasis, thereby offering significant promise for the treatment of IBD.

## Experimental status of BA-targeted therapeutic strategies

### Microbiota-based therapy

Fecal microbiota transplantation (FMT) is a therapeutic approach aimed at restoring gut microbial homeostasis. It was initially established as an effective treatment for recurrent *Clostridioides difficile* infection,^90^ with its efficacy largely attributed to the restoration of microbial BA metabolism, particularly the generation of bile salt hydrolase (BSH)-dependent SBAs such as DCA and LCA.^91^

These SBAs not only inhibit the growth and spore germination of *C. difficile* but are also thought to play an important role in immune regulation in IBD. In IBD, including CD and UC, the clinical outcomes of FMT have been variable. Some clinical trials have indicated that FMT can alleviate symptoms in patients with active UC and improve endoscopic severity indices. Further studies suggest that the therapeutic effect correlates with the engraftment of specific microbial taxa. Metagenomic analyses indicate that the restoration of microbially derived SBAs, such as 3-oxoLCA, following FMT, correlates with clinical remission, underscoring the central role of BA metabolism in the mechanism of FMT.^92^

However, FMT has important limitations. The process of transferring an entire microbial community lacks specificity and may introduce inflammation-associated bacteria (e.g., certain proteobacteria), leading to inconsistent outcomes or potential adverse effects. Therefore, future therapeutic strategies should move beyond whole-microbiome replacement toward more targeted microbial interventions. Developing defined bacterial consortia with specific BA-metabolizing capabilities (e.g., high BSH activity) could enable more precise modulation of the gut microbiome, potentially restoring Th17/Treg balance with improved efficacy and safety.

### Probiotic supplementation

Probiotics represent a promising microecological intervention for IBD through modulation of the gut microbial community. Compared with traditional FMT, probiotic approaches offer greater strain specificity and mechanistic clarity, demonstrating distinct advantages in the regulation of BA metabolism.

Probiotics with specific BA-metabolizing capabilities can precisely reshape the composition of the BA pool under disease conditions. For instance, *Clostridium* species possessing 7α-dehydroxylase activity produce SBAs such as DCA and LCA, which inhibit the growth of pathogens like *Clostridioides difficile*.^93^ Recent genetic engineering approaches have enabled introduction of the BA-inducible operon into commensal bacteria such as *Clostridium sporogenes*, granting them the ability to synthesize specific SBAs and opening new avenues for precise manipulation of the gut environment.^94^ Furthermore, commonly used probiotic formulations like VSL#3 and *Lactobacillus reuteri* NCIMB 30242 are rich in BSH-expressing strains. These strains promote the deconjugation of BAs, thereby influencing signaling through BA receptors such as FXR and PXR.^95^^,^^96^ In animal studies, VSL#3 has been shown to alleviate colitis and suppress tumorigenesis, which are associated with the restoration of FXR/PXR signaling and the modulation of Th17/Treg homeostasis.^97^^,^^98^

Beyond direct BA modulation, certain probiotics (e.g., *Bifidobacterium adolescentis* and *Escherichia coli Nissle* 1917) and their engineered derivatives can enhance antioxidant capacity and repair the intestinal epithelial barrier, indirectly ameliorating IBD pathology.^99^ Prebiotics like inulin can synergize with probiotics by promoting butyrate production and suppressing pathogenic bacteria.^100^

However, the efficacy of probiotics is strain specific and context dependent, potentially causing intolerance or variable responses in some patients. Therefore, future efforts should focus on screening next-generation probiotics with efficient and stable BA-metabolizing functions and elucidating their immunomodulatory mechanisms in restoring Th17/Treg balance to advance personalized probiotic therapies for IBD.

### Conventional BA modulators

The direct administration of bioactive SBAs represents a promising therapeutic strategy for IBD. Compared to interventions that introduce entire microbial communities, this approach offers a more direct mechanism of action and greater target specificity, demonstrating unique potential for modulating Th17/Treg immune homeostasis (Table 2).

**Table 2:** Experiment status of bile acid-related treatment strategies^a^

Treatment category	Specific intervention	Mechanism of BA modulation	Experimental evidence
Microbiota-based therapy	Fecal microbiota transplantation	restores gut microbial diversity; restores bile acid metabolism of the gut microbiota (increases secondary BAs like DCA/LCA); induction of immune tolerance; corrects bile acid absorption disorder	peak clinical response and remission rates of 86.7% and 76.7%, respectively, were observed at 1 month post-FMT in a study of 30 refractory CD patients^121^; in the largest and longest-follow-up study to date involving 174 CD patients, 75.3% achieved clinical response at 1 month post-FMT, while the clinical remission rate was only 20.1% at a median follow-up of 43 months.^122^
Probiotic supplementation	*Bifidobacterium* + *Lactobacillus* combinations	produce bile salt hydrolase; promote production of beneficial metabolites; restore microbial-bile acid axis	high-dose probiotics induced clinical remission in 77% vs. 33% of patients and elevated butyrate levels, which inversely correlated with disease activity.^100^
Conventional BA modulators	Bile acid sequestrants	bind excess primary bile acids in intestine; modulate Th17/Treg balance	in AOM/DSS-induced CRC model, UDCA inhibited tumor growth in a concentration-dependent manner and decreased the expression of YAP and Ki67.^102^
BA receptor agonists	FXR agonists (e.g., obeticholic acid)	regulate bile acid synthesis; reduce primary bile acid overproduction; strengthen intestinal barrier	INT-747 alleviated murine colitis by improving epithelial barrier function and suppressing pro-inflammatory cytokines (TNF-α and IL-1β).^106^
	GPBAR1 agonists (e.g., INT-777)	enhance anti-inflammatory responses; promote epithelial repair; modulate Th17/Treg balance	INT-777 improved metabolic and gut homeostasis by promoting GLP-1 secretion and reducing inflammation.^109^

a Abbreviation: BAs, Bile acids; FMT, Fecal microbiota transplantation; CD, Crohn’s disease; BSH, Bile salt hydrolase; DCA, Deoxycholic acid; LCA, Lithocholic acid; Treg, Regulatory T cell; Th17, T helper 17 cell; AOM/DSS, Azoxymethane/Dextran sulfate sodium; CRC, Colorectal cancer; UDCA, Ursodeoxycholic acid; YAP, Yes-associated protein; FXR, Farnesoid X receptor; TNF-α, Tumor necrosis factor-alpha; IL-1β, Interleukin-1 beta; GPBAR1, G protein-coupled bile acid receptor 1; GLP-1, Glucagon-like peptide-1.

Among the BAs studied, UDCA and LCA have garnered significant attention. UDCA, a classic hepatoprotective agent, is widely used for autoimmune cholestatic liver diseases such as primary biliary cholangitis.^101^ Research indicates that UDCA can inhibit colorectal cancer progression via activation of the membrane receptor GPBAR1, a process associated with sex-specific alterations in gut microbiota composition.^102^ More importantly, LCA and its metabolites have been found to promote the differentiation of naive T cells into anti-inflammatory Tregs while suppressing the pro-inflammatory Th17 cell pathway. This occurs through modulation of the activity of transcription factor RORγt and mitochondrial function. This mechanism has been validated in animal models, effectively reducing colitis severity and partially explaining the efficacy of certain microbial therapies.^64^

From a molecular perspective, both UDCA and LCA can act as agonists for FXR. Activation of the FXR signaling pathway is crucial not only for maintaining BA and metabolic homeostasis but also plays a central role in alleviating metabolic syndrome and related inflammation through the downstream FXR-FGF15/19 axis.^103^ Consequently, the synthetic FXR agonist obeticholic acid has been approved for clinical use and has shown efficacy in treating conditions like non-alcoholic steatohepatitis, highlighting the potential value of modulating this pathway in IBD management.^104^

However, the therapeutic effects of direct BA supplementation may be transient, limited primarily to the treatment period, with challenges in maintaining efficacy after discontinuation. Therefore, future research may focus on combining direct supplementation with microbiota modulation strategies. Utilizing specific probiotics or synbiotics to persistently optimize the host’s intrinsic BA metabolic network could achieve long-term, stable regulation of Th17/Treg balance, offering a more fundamental therapeutic option for IBD patients.

### BA receptor agonists

FXR, a key NR for BAs, plays a central role in maintaining intestinal barrier integrity, inhibiting bacterial translocation, and regulating immune responses, making it a promising therapeutic target for IBD.^105^ Obeticholic acid (INT-747), a highly selective FXR agonist, has demonstrated strong anti-colitis effects in preclinical studies. It ameliorated chemically induced colitis in mice by improving epithelial permeability, reducing goblet cell loss, and suppressing pro-inflammatory cytokines such as TNF-α and IL-1β.^106^

Beyond obeticholic acid, next-generation FXR agonists are under active development. Molecular dynamics simulations have revealed that ligand interactions with the FXR ligand-binding domain—particularly the H8 helix—critically influence agonist potency, guiding rational drug design.^107^ Non-steroidal FXR agonists like cilofexor and tropifexor are being evaluated in clinical trials for non-alcoholic steatohepatitis, with their potential in IBD under exploration.^108^ Agonists targeting other BA receptors, such as the GPBAR1 agonist INT-777, also show promise by promoting GLP-1 secretion and reducing inflammation.^109^

Despite promising preclinical results, the clinical use of FXR agonists in IBD remains in early stages. Limited trials have been conducted, and long-term efficacy, safety, and patient-specific responses require further validation. Systemic FXR activation may cause side effects like pruritus and dyslipidemia, motivating the development of gut-restricted FXR agonists to enhance local efficacy while minimizing systemic exposure. A deeper understanding of how FXR signaling regulates Th17/Treg balance will be essential for advancing targeted BA-based therapies for IBD.

## Conclusions

In recent years, the incidence of IBD has been on a steady rise globally, drawing increasing attention to the intricate interplay between intestinal microbiota, BA metabolism, and disease pathogenesis. Mounting evidence confirms that both gut microbial dysbiosis and disrupted BA metabolism are key drivers of IBD development and progression—dysregulated microbiota alters BA synthesis and transformation, while impaired BA metabolism disturbs intestinal immune homeostasis, creating a vicious cycle that exacerbates inflammation.

To improve IBD prevention and treatment, interventions must target these underlying mechanisms. Conventional approaches like antibiotic therapy can mitigate pathogenic microbial overgrowth but require careful use to avoid further disruption of the microbiome. BA receptor modulators offer promise by restoring BA signaling and immune regulation. Probiotics and prebiotics help rebalance the microbiota, enhancing beneficial BA-producing bacteria. FMT directly replenishes functional microbial communities, correcting BA metabolism defects. Emerging strategies such as phage therapy provide targeted suppression of harmful microbes without damaging commensals.

Future efforts should focus on combining these approaches, tailored to individual microbial and BA profiles, to develop precise, mechanism-based therapies. By integrating insights into the microbiota-BA-immune axis, we can move beyond symptomatic control toward more effective prevention and curative strategies for IBD.

## Acknowledgments

We would like to extend our heartfelt thanks to the Central Laboratory of Renmin Hospital, Wuhan University for their technical support. We also thank L.Z. Li for supporting this study. This work was supported by the Fundamental Research Funds for the Central Universities (2042024YXA002), Shenzhen Science and Technology Program (JCYJ20220530140609019 and JCYJ20230807090205011), and the Interdisciplinary Innovative Talents Foundation from Renmin Hospital of Wuhan University (JCRCZN-2022-018).

## Author contributions

Conceptualization, S.Y.; writing – original draft and writing – review & editing, S.Y., L.G., and Y.T.; visualization, F.L. All authors have read and agree to the published version of the manuscript.

## Declaration of interests

The authors declare no competing interests.
